# Telmisartan, when compared to losartan, shows additional beneficial effects to pancreatic islet structure and function in diet-induced obese mice

**DOI:** 10.1186/1758-5996-7-S1-A120

**Published:** 2015-11-11

**Authors:** Francielle Graus-Nunes, Felipe de Olivera Santos, Thatiany de Souza Marinho, D'Angelo Carlo Magliano, Fernanda Ornellas, Marcia Barbosa Aguila, Carlos Alberto Mandarim de Lacerda, Vanessa de Souza Mello

**Affiliations:** 1 Universidade do Estado do Rio de Janeiro, Rio de Janeiro, Brazil

## Background

In animal studies, telmisartan elicits weight loss by activation of the peroxisome proliferator-activated receptors (PPARs), besides blocking AT1-r downstream effects. The effects of telmisartan upon pancreatic islets remain to be unraveled.

## Objective

We sought to evaluate the effects of telmisartan (AT1-r blocker and PPAR agonist) or losartan (pure AT1-r blocker) on pancreatic islet structure and function in a diet-induced obesity mice model.

## Materials and methods

C57BL/6 mice were fed a standard chow (SC; 10% lipids) or a high-fat diet (HF; 50% lipids) for 10 weeks. Then, animals were randomly allocated into six groups to start the treatment with Telmisartan (T) or Losartan (L): SC, SC-L, SC-T, HF, HF-L and HF-T. The treatment phase lasted 5 weeks. Pair feeding (PF) groups were carried out to telmisartan treated groups (SCT-PF and HFT-PF) so as to isolate the effects of the treatment from the effects of reduced diet intake upon the evaluated parameters. Differences among the groups were tested with One-way ANOVA and Holm-Sidak post hoc test (P<0.05).

## Results

HFT had lower energy intake and body mass (BM) at the end of treatment than HF and HFL. HFL group had energy intake and BM similar to HF. HFT-PF revealed that BM reduction in HFT can be exclusively attributed to telmisartan as HFT-PF ate the same amount of food as HFT, but did not exhibit reduced BM in comparison to untreated HF. HFT group showed reduced fasting glucose levels when compared to HF, which was not found in HFL group. However, regarding oral glucose tolerance test and insulin levels, both parameters were improved by telmisartan and losartan when compared to HF. Regarding pancreatic islet architecture and function, HFT and HFL groups had smaller islet mass and β–cell mass than HF. The same behavior was observed for α-cell in both treated groups. However, HFT showed the smallest values of α-cell, with no difference to SC. Interestingly, a qualitative increase in islet vascularization from both treated groups was observed after alpha-smooth muscle actin immunostaining in confocal microscopy.

## Conclusions

Telmisartan treatment yielded improvement in all metabolic parameters, besides beneficial pancreatic islet remodeling and function, suggesting that PPAR activation may mediate these additional effects when compared to losartan. Enhanced blood flow to the islets might also be implicated into amelioration of islet structure and function in obese treated mice.

